# Aspirin as a potential modality for the chemoprevention of breast cancer: A dose-response meta-analysis of cohort studies from 857,831 participants

**DOI:** 10.18632/oncotarget.16315

**Published:** 2017-03-17

**Authors:** Liming Lu, Leiyu Shi, Jingchun Zeng, Zehuai Wen

**Affiliations:** ^1^ The Second Affiliated Hospital of Guangzhou University of Chinese Medicine, Guangdong Provincial Hospital of Chinese Medicine, Guangzhou, China; ^2^ Department of Health Policy and Management, Bloomberg School of Public Health, Johns Hopkins University, Baltimore, MD, USA; ^3^ Guangzhou University of Chinese Medicine, Guangzhou, China; ^4^ National Center for Design Measurement and Evaluation in Clinical Research, Guangzhou University of Chinese Medicine, Guangzhou, China

**Keywords:** aspirin, breast cancer, dose-response Meta-analysis

## Abstract

**Background:**

Previous meta-analyses on the relationship between aspirin use and breast cancer risk have drawn inconsistent results. In addition, the threshold effect of different doses, frequencies and durations of aspirin use in preventing breast cancer have yet to be established.

**Results:**

The search yielded 13 prospective cohort studies (N=857,831 participants) that reported an average of 7.6 cases/1,000 person-years of breast cancer during a follow-up period of from 4.4 to 14 years. With a random effects model, a borderline significant inverse association was observed between overall aspirin use and breast cancer risk, with a summarized RR = 0.94 (*P* = 0.051, 95% CI 0.87-1.01). The linear regression model was a better fit for the dose-response relationship, which displayed a potential relationship between the frequency of aspirin use and breast cancer risk (RR = 0.97, 0.95 and 0.90 for 5, 10 and 20 times/week aspirin use, respectively). It was also a better fit for the duration of aspirin use and breast cancer risk (RR = 0.86, 0.73 and 0.54 for 5, 10 and 20 years of aspirin use).

**Methods:**

We searched MEDLINE, EMBASE and CENTRAL databases through early October 2016 for relevant prospective cohort studies of aspirin use and breast cancer risk. Meta-analysis of relative risks (RR) estimates associated with aspirin intake were presented by fixed or random effects models. The dose-response meta-analysis was performed by linear trend regression and restricted cubic spline regression.

**Conclusion:**

Our study confirmed a dose-response relationship between aspirin use and breast cancer risk. For clinical prevention, long term (>5 years) consistent use (2-7 times/week) of aspirin appears to be more effective in achieving a protective effect against breast cancer.

## INTRODUCTION

Worldwide, breast cancer comprises 23% of total cancer cases and causes 14% of cancer related deaths in women [1]. There are many risk factors for breast cancer including environmental, hereditary, dietary and socioeconomic. However, the underlying mechanism behind breast cancer is not fully understood [2, 3].

Aspirin may inhibit the cancer-initiating stage through the cyclooxygenase (COX) enzyme system. The production of prostaglandins, cyclic adenosine monophosphate (cAMP), aromatase activity and cell proliferation in breast cancer cells are stimulated by COX-2. Aspirin may block the COX-2 enzyme to inhibit this process [4]. Therefore, aspirin is a potential agent for the chemoprevention of breast cancer. Furthermore, it has been confirmed that aspirin use is associated with the risk of colorectal, ovarian and prostate cancer [5–8].

However, several epidemiological studies assessing the relationship between breast cancer risk and aspirin use have drawn inconsistent conclusions [5–8]. The frequency-risk, dose-risk and duration-risk relationships still need to be explored. Recently, several large cohort studies have explored the relationship between aspirin intake and breast cancer risk [9, 10]. A sufficient number of cohort studies have been conducted in recent years, which have also benefited our probe of a dose-response relationship. This study aims to 1) test whether there is a dose-response relationship between aspirin use and breast cancer risk; 2) confirms the optimal frequency, duration and dose of aspirin intake in preventing breast cancer, and guide the rational application of aspirin in clinic.

## RESULTS

### Literature search

**Figure 1:**
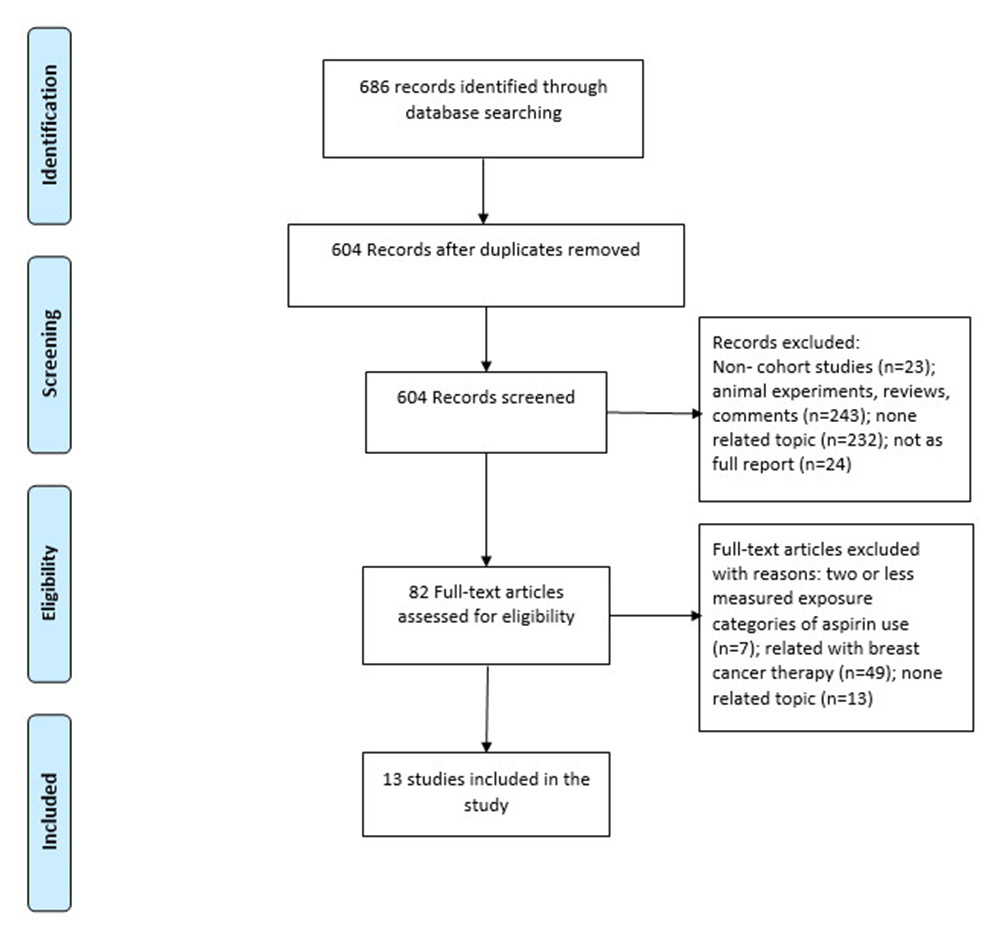
Flow chart of reports selection

686 unique citations were retrieved: 441 from Medline, 148 from Embase and 97 from CENTRAL. Of these, 604 were excluded after identification, screening and eligibility processes, based on titles or abstracts, leaving 82 citations for full-text review. Of the 82 remaining citations, 69 were excluded and 13 were included. The article selection process is presented in Figure 1.

### Characteristics

Characteristics of the 13 selected cohort studies are presented in Table 1. Included studies were published between 2002 and 2015, and the number of participants per study ranged from 23,708 to 127,383, for a total of 857,831 (average 7.6 cases/1,000 person-years of breast cancer). 10 studies were conducted in the United States, 1 in the Netherlands, 1 in Denmark and 1 in the United Kingdom. Breast cancer was screened along with medical records and pathology reports, or through linkages with cancer registries in 12 studies (one study had no report of this). Aspirin use was measured from self-reported data in 10 studies, and computer assisted telephone interviews in 1 study (1 study had no reporting of this). Follow-up duration ranged from 4.4 to 14 years. 11 of those studies had good quality with an NOS score ≥7, while only two studies’ NOS ratings (Harris RE-2003 [33] and Kim S-2015 [16]) were less than 7.

**Table 1:** Characteristics of studies included in our study

Source	Location	Cohort Designation	Total No. of Patients	Breast cancer incidence (Cases/1000 Person-Years)	Aspirin Use Measure and assessment periods	Breast Cancer Screening Method	Maximum Follow-up, y	NOS
Jacobs EJ-2005 [11]	United States	Cancer Prevention Study II Nutrition Cohort	77413	5.5	Patient reported: dose, frequency, duration; baseline and follow-up	Initially by patient report, follow-up by questionnaires and subsequently verified medical record or through linkage with state registries	10	8
Johnson TW-2002 [12]	United States	Iowa Women's Health Study	27616	4.9	Patient reported: frequency; baseline	Breast cancer incidence was ascertained by linkage to the State Health Registry of Iowa	6	8
Harris RE-2003 [13]	United States	Women's Health Initiative Observational Study	80741	44.81	Patient reported: duration; baseline	Initially by patient report, Potential cases were identified through the annual follow-up questionnaires or from nonroutine contacts; all examination reports were reviewed	NA	6
Bardia A-2011 [14]	United States	Iowa Women's Health Study	26580	5.1	Patient reported: frequency; follow-up	Identified by linking to the Iowa Cancer Registry	13	7
Hollestein LM-2014 [9]	Netherlands	Eindhoven Cancer Registry and the PHARMO Record Linkage System	109276	10.5	NA; duration; baseline and follow-up	NA	4.4	7
Kim S-2015 [10]	United States	Sister Study	50884	NA	CATI-based self-reported: frequency, duration; baseline	By pathology reports or complete medical report	10	6
Friis S-2008 [15]	Denmark	Danish Diet, Cancer and Health cohort study	28695	3.9	Patient reported: frequency, duration; baseline and follow-up	Information on cancer diagnoses was obtained from the Danish Cancer Registry	10	8
Gierach GL-2008 [16]	United States	National Institutes of Health-AARP Diet and Health Study	127383	5.3	Patient reported: frequency; baseline	Initially identified through probabilistic linkage to eight state cancer registries; the cancer registry ascertainment area was recently expanded to include three additional states to capture cancer occurrence	7.13	8
Gill JK-2007 [17]	United States	Multiethnic Cohort	98920	NA	Patient reported: duration; baseline	Identified through linkages with the Los Angeles County Cancer Surveillance Program, the State of California Cancer Registry, and the Hawaii Tumor Registry	9	8
Ready A-2008 [18]	United States	Vitamins And Lifestyle study	35323	NA	Patient reported: dose; baseline	Ascertained through annual linkage of the VITAL cohort database to the SEER cancer registry	10	8
Bosco JL-2011 [19]	United States	Black Women's Health Study	59000	2.3	Patient reported: duration; baseline and follow-up	Reported on follow-up questionnaires; to date, medical records or cancer registry data have been obtained for 99.4% of reported cases	12	7
García Rodríguez LA-2004 [20]	United Kingdom	General Practice Research Database	23708	15.6	NA; dose, duration; baseline	Identified patients with a code of breast cancer and manually reviewed their computerized patient profiles	6	8
Eliassen AH-2009 [21]	United States	Prospective Nurses’ Health Study II	112292	1.1	Patient reported: frequency, duration; baseline and follow-up	Questionnaires and medical records	14	8

Abbreviations: NA, not recorded or available; CATI, computer assisted telephone interview.

Most risk measures were adjusted for age (12 studies), health history (9 studies), body mass index (BMI) (9 studies), education (6 studies), use of hormone therapy (6 studies) or alcohol consumption (6 studies); less were adjusted for mammography (5 studies), smoking (3 studies), non-steroidal anti-inflammatory drug (NSAIDs) use (4 studies), physical activity (3 studies), contraceptive use (3 studies) or weight (3 studies). (Table 2).

**Table 2:** Confounding factors and methods for adjustment

Source	Method for adjustment	Risk expression	Confounding factors
Jacobs EJ-2005 [11]	Cox proportional hazards model	RR	Age, race, education, family history of breast cancer, personal history of breast cysts, history of mammography, age at menarche, duration of oral contraceptive use, parity, age at menopause, use of hormone replacement therapy, weight change, BMI, alcohol consumption and duration of use of other NSAID types
Johnson TW-2002 [12]	Multivariate adjustment	RR	Age, BMI, estrogen use, family history of breast cancer, benign mammary disease, multivitamin use, NSAID use, mammography, and waist: hip ratio
Harris RE-2003 [13]	Cox multivariate regression	RR	Age
Bardia A-2011 [14]	Cox proportional hazards models	RR	Age, education, family history of breast cancer, age at menarche, age at menopause, parity/age at first live birth, use of oral contraceptives, use of hormone therapy, BMI in 1992, BMI at age 18 years, relative weight at age 12, history of osteoarthritis, history of rheumatoid arthritis, smoking, use of alcohol and physical activity level
Hollestein LM-2014 [9]	Cox proportional hazard model	HR	Age, sex, unique number of dispensings and unique number of hospitalizations
Kim S-2015 [10]	Cox regression model	HR	Race/ethnicity, level of education, history of benign proliferative mammary disease, number of 1st degree family members with breast cancer, BMI, age at 1st term birth, time since the last mammogram and menopause status at diagnosis
Friis S-2008 [15]	Cox proportional hazards regression	RR	Age, school education, parity, number of births, use of hormone replacement therapy and history of benign mammary tumor surgery
Gierach GL-2008 [16]	Proportional hazards model	RR	Age, race, age at first birth, hormone therapy use, number of breast biopsies, alcohol intake, history of hypertension, and family history of breast cancer in first-degree relative
Gill JK-2007 [17]	Multivariate Cox proportional hazards model	HR	Age, ethnicity, BMI, family history of breast cancer, education, mammography screening, alcohol intake, age at menarche, age at first live birth, number of children, menopausal status, age at menopause, and hormone replacement therapy
Ready A-2008 [18]	Cox proportional hazards model	HR	Age, race, BMI, family history of breast cancer, history of breast biopsy, mammogram within 2 years prior to baseline, age at menarche, age at first birth, age at menopause, history of surgical menopause, years of combined estrogen and progesterone hormone therapy, multivitamin use and alcohol use and use of other categories of NSAIDs
Bosco JL-2011 [19]	Cox proportional hazards regression	RR	Education, BMI at age 18, vigorous activity, female hormone use, smoking and other NSAIDs
García Rodríguez LA-2004 [20]	Multivariate adjustment	OR	Age, calendar year, BMI, alcohol intake, smoking status, use of hormone replacement therapy, prior benign mammary disease, and others
Eliassen AH-2009 [21]	Cox proportional hazards models	RR	Age at menarche, height, BMI at age 18, weight change since age 18, oral contraceptive use, parity and age at first birth, alcohol consumption, history of benign breast disease, family history of breast cancer

Abbreviations: RR: relative risk; HR: hazard ratios; OR: odd ratio; BMI: body mass index; NSAIDs: non-steroidal anti-inflammatory drugs.

### Comparisons of different dosages, frequencies and durations of aspirin intake

**Figure 2:**
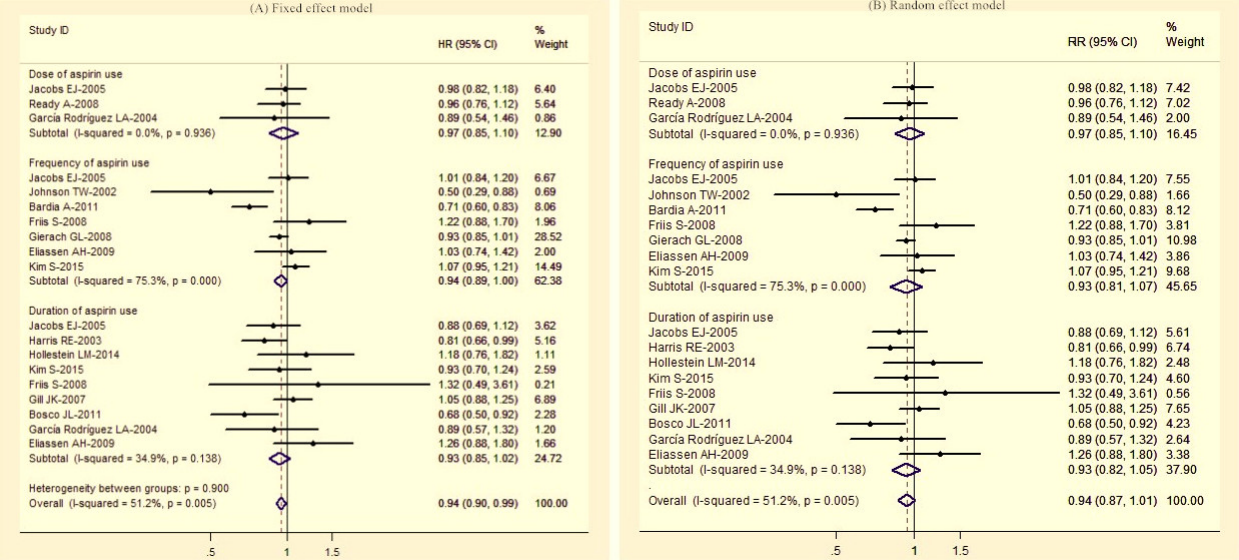
Relative risk of colorectal cancer for highest vs. lowest categories of aspirin use (dose, frequency and duration)

The overall RRs of breast cancer with the highest, compared with the lowest dosages, duration, and frequency are shown in Figure 2A. There was no heterogeneity among studies of dosage (*P* = 0.936, *I^2^*= 0.0%) or duration (*P* = 0.138, *I^2^*= 34.9%); but significant heterogeneity was observed for frequency (*P* = 0.000, *I^2^*= 75.3%) and the overall usage of aspirin (*P* = 0.005, *I^2^*= 51.2%). With fixed effects models, no evidence of breast cancer risk was found in frequency (RR = 0.94, 95% CI 0.89–1.00), dosage (RR = 0.97, 95% CI 0.85–1.10), or duration (RR = 0.93, 95% CI 0.85–1.02) of aspirin used. These results were similar when a random effects model was applied: frequency (RR = 0.93, 95% CI 0.81–1.07), dose (RR = 0.97, 95% CI 0.85–1.10) and duration (RR = 0.93, 95% CI 0.82–1.05) of aspirin intake (Figure 2B).

With a random effects model, a borderline inverse relationship was observed between overall aspirin intake (including dosage, frequency and duration) and breast cancer risk, with summarized RR = 0.94 (*P* = 0.051, 95% CI 0.87–1.01) (Figure 2B). This result was similar with that of the fixed effects model (RR = 0.94, 95% CI 0.90–0.99) (Figure 2A).

### Dose-response analysis

As the number of included studies related to aspirin dosage and breast cancer risk was small (Table 3), and the information on breast cancer incidence (cases/1,000 person-years) was missing in the study of Ready A-2008 [18] (Table 1), dose-response analysis could not be performed for aspirin dose and breast cancer risk.

**Table 3:** Epidemiological studies of aspirin dose (mg/week) and breast cancer

Author, year	Aspirin dose (mg/daily)	Dose midpoint (mg/daily)	RR	95% CI for RR
Jacobs EJ-2005 [11]	0	0	1	Reference
	1-325	162.5	1.11	1.01-1.22
	>325	390	0.98	0.82-1.18
Ready A-2008 [18]	0	0	1	Reference
	1-325	162.5	0.99	0.80-1.23
	>325	390	0.96	0.76-1.22
García Rodríguez LA-2004 [20]	0	0	1	Reference
	75	75	0.67	0.51-0.89
	150	150	0.96	0.65-1.41
	300	300	0.89	0.54-1.46

Abbreviations: RR: relative risk; CI: confidence intervals.

**Figure 3:**
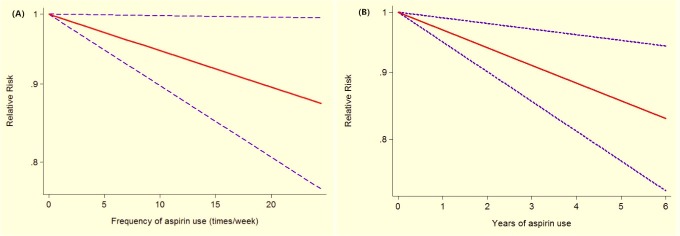
Association between frequency (years) of aspirin use and risk of breast cancer obtained by linear dose-response meta-analyses

Studies of aspirin intake frequency (times/week) and breast cancer are presented in Table 4. The non-linear association of frequency and breast cancer risk had no significance in the spline model (*P* = 0.275). Therefore, a linear regression was built (*P* = 0.041; Figure 3A). Breast cancer risk decreased as aspirin intake frequency increased. Breast cancer risk for 5 times/week aspirin intake was 0.97 (95% CI 0.95–0.99). There was a trend of decreasing risk along with higher aspirin intake frequency (RR = 0.95, 95% CI 0.90–0.99, for 10 times/week and RR = 0.90, 95% CI 0.81–0.99, for 20 times/week).

**Table 4:** Epidemiological studies of frequency of aspirin use (times/week) and breast cancer

Author, year	Frequency of aspirin use(times/week)	Frequency midpoint(times/week)	RR	95% CI for RR
Jacobs EJ-2005 [11]	0	0	1	Reference
	0.25-3.50	1.88	1.14	1.04-1.25
	3.75-7.25	5.50	0.89	0.76-1.03
	7.50-14.75	11.13	0.96	0.85-1.09
	≥15	18.0	1.01	0.84-1.20
Johnson TW-2002 [12]	0	0	1	Reference
	<1	0.50	1.10	0.72-1.67
	1	1	1.71	0.93-3.13
	2-5	3.50	0.97	0.59-1.58
	**≥6**	7.20	0.50	0.29-0.88
Bardia A-2011 [14]	0	0	1	Reference
	≤1	0.50	0.87	0.76-0.99
	2-5	3.50	0.78	0.66-0.92
	≥6	7.20	0.71	0.60-0.83
Friis S-2008 [15]	0	0	1	Reference
	0.50-0.75	0.63	1.25	1.02-1.53
	1.0-6.0	3.50	1.45	1.16-1.82
	7.0-42.0	24.50	1.22	0.88-1.70
Gierach GL-2008 [16]	0	0	1	Reference
	<1	0.50	0.95	0.89-1.03
	1-6	3.50	0.95	0.87-1.04
	≥7	8.40	0.93	0.85-1.01
Kim S-2015 [10]	0	0	1	Reference
	<4	2	0.87	0.68-1.13
	4-6	5	1.14	0.90-1.43
	≥7	8.4	1.07	0.95-1.21
Eliassen AH-2009 [21]	0	0	1	Reference
	1	0.50	1.01	0.78-1.30
	2-3	2.50	1.18	0.87-1.60
	4-5	4.50	0.64	0.35-1.16
	≥6	7.20	1.03	0.74-1.42

Abbreviations: RR: relative risk; CI: confidence intervals.

Studies of aspirin intake years and breast cancer are presented in Table 5. The non-linear association of years of aspirin intake and breast cancer risk was significant in the cubic spline model (*P* = 0.014). However, a nonlinearity (*P* = 0.305) test did not refute the null hypothesis the linear model was available. Thus, a linear regression was conducted (*P* = 0.004; Figure 3B). Breast cancer risk decreased as years of aspirin intake increased. If aspirin had been taken for 5 years, breast cancer risk was 0.86 (95% CI 0.77–0.95). There was a trend of decreasing risk for more years of aspirin intake (RR = 0.73, 95% CI 0.59–0.91, for 10 years and RR = 0.54, 95% CI 0.35–0.82, for 20 years). Table 5 shows that most of the included studies reported that the aspirin intake frequency was ≥2 times per week for each duration.

**Table 5:** Epidemiological studies of years of aspirin use and breast cancer

Author, year	Years of aspirin use	Frequency or dose of aspirin use	Years midpoint	RR	95% CI for RR
Jacobs EJ-2005 [11]	0	0	0	1	Reference
	<5	≥1 pills per day	2.50	1.08	0.94-1.23
	≥5	≥1 pills per day	6.0	0.88	0.69-1.12
Harris RE-2003 [13]	0-0.92	two or more tablets/week	0.46	1	Reference
	1-4	two or more tablets/week	2.5	0.90	0.72-1.13
	≥5	two or more tablets/week	6	0.81	0.66-0.99
Hollestein LM-2014 [9]	<2	≤100 mg daily	1	1	Reference
	2-6	≤100 mg daily	4	1.12	0.90-1.40
	>6	≤100 mg daily	7.2	1.18	0.76-1.82
Kim S-2015 [10]	<5	≥2 times per week	2.5	1	Reference
	5-9	≥2 times per week	7.5	0.83	0.69-1.02
	10-20	≥2 times per week	15	0.95	0.76-1.19
	>20	≥2 times per week	24	0.93	0.70-1.24
Friis S-2008 [15]	0	0	0	1	Reference
	<1	≥1 pills per month	0.5	1.26	0.85-1.87
	1-3	≥1 pills per month	2	1.14	0.60-2.15
	≥4	≥1 pills per month	4.8	1.32	0.49-3.61
Gill JK-2007 [17]	0	0	0	1	Reference
	≤1	≥2 times per week	0.5	1.01	0.79-1.30
	2-5	≥2 times per week	3.5	0.89	0.72-1.09
	≥6	≥2 times per week	7.2	1.05	0.88-1.25
Bosco JL-2011 [19]	0	absence	0	1	Reference
	<1	absence	0.5	0.84	0.41-1.70
	1-1.9	absence	1.5	0.37	0.05-2.66
	2-2.9	absence	2.5	0.89	0.68-1.16
	3-4.9	absence	4	0.84	0.59-1.19
	≥5	absence	6	0.68	0.50-0.92
García Rodríguez LA-2004 [20]	0	0	0	1	Reference
	0-0.9	75-300 mg daily	0.45	0.89	0.70-1.13
	1-1.9	75-300 mg daily	1.45	0.69	0.42-1.10
	2-3.9	75-300 mg daily	2.95	0.90	0.64-1.24
	≥4	75-300 mg daily	4.8	0.89	0.57-1.32
Eliassen AH-2009 [21]	0	0	0	1	Reference
	<5	≥2 times per week	2.5	1.03	0.84-1.26
	≥5	≥2 times per week	6	1.26	0.88-1.80

Abbreviations: RR: relative risk; CI: confidence intervals; 1 pill or tablet was equal to 325 mg.

### Sensitivity analysis

**Figure 4:**
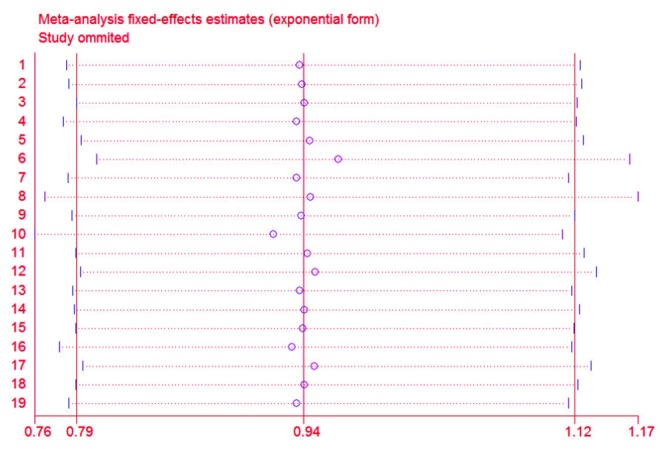
Sensitivity analyses through exclusion of 1 study at a time to reflect the influence of individual study to the overall results

Sensitivity analyses conducted by excluding one study at a time indicated that each individual dataset had no significant influence on the overall results (Figure 4). An alternative sensitivity analysis based on those studies with a score of 7 or more was also conducted. When two studies with NOS ratings less than 7 (Harris RE-2003 [13] and Kim S-2015 [10]) were excluded, the pooled overall RR was 0.93 (95% CI: 0.86–1.13), and the RR for frequency was 0.90 (95% CI: 0.76-1.06) for the remaining studies.

We have summarized the assessment stage of aspirin exposure (e.g. at the cohorts' baselines, during follow-up periods or at both stages) in Table 1. We also performed sensitivity analysis by combining studies at different assessment stages of aspirin exposure, and found the results similar.

### Publication bias

**Figure 5:**
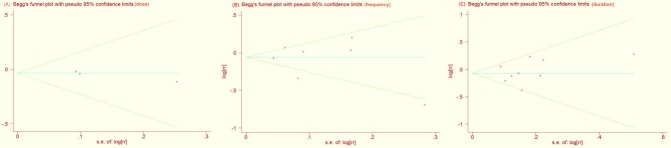
Begg's funnel plot with 95% confidence limits to detect publication bias (dose, frequency and duration)

Funnel plots and statistical tests showed no publication bias for included reports of dosage (Figure 5A; Egger's test *P*= 0.227; Begg's test *P*= 0.296) or frequency (Figure 5B; Egger's test *P*= 0.755; Begg's test *P*= 1.000) and duration (Figure 5C; Egger's test *P*= 0.612; Begg's test *P*= 0.602).

## DISCUSSION

Many epidemiologic studies exploring the association between breast cancer risk and aspirin intake have inferred inconsistent results. To obtain a better understanding of this issue, several meta-analyses have been published in recent years. Takkouche B [6] has drawn the conclusion that aspirin is related to decreased risk for breast cancer. Zhao YS [7] found that aspirin intake is related to a slight decrease in the development of breast cancer with a marginally statistically significant difference. Mangiapane S [5] found that aspirin might decrease breast cancer risk. However, these results must be interpreted carefully, as exposure categories have been defined heterogeneously among the studies which have weakened the validity of the pooled estimates. In addition, Mangiapane S [5] and Jacobo-Herrera NJ [22] have pointed out that the relationship between breast cancer and aspirin, dosage, time and frequency of aspirin use have yet to be established. Therefore, it is necessary to perform this dose-response meta-analysis with cohort design to offer a more confident and exhaustive solution to a possible inverse association between breast cancer risk and aspirin intake [22].

Our study confirmed a dose-response relationship between aspirin use and breast cancer risk. Our findings have demonstrated that a borderline significant inverse association exists between breast cancer risk and overall aspirin intake (RR = 0.94; *P* = 0.051; 95% CI: 0.87–1.01). Additionally, the linear dose-response analysis displayed a potential association between the aspirin intake frequency and breast cancer risk (RR = 0.97 for 5 times/week aspirin use; RR = 0.95 for 10 times/week aspirin use; RR = 0.90 for 20 times/week), and also for the duration of aspirin intake and breast cancer risk (RR = 0.86 for 5 years of aspirin use; RR = 0.73 for 10 years of aspirin use; RR = 0.54 for 20 years of aspirin use). We first comprehensively and quantitatively evaluated these relationships.

In regards to implications for clinical prevention, firstly an innovative finding from this research is the existence of a threshold effect between aspirin intake and breast cancer risk. Note that 5, 10 and 20 times/week aspirin use only reduced 3% (1-0.97), 5% (1-0.95) and 10% (1-0.90) of breast cancer risk; 5, 10 and 20 years of aspirin use can reduce 14% (1-0.86), 27% (1-0.73) and 46% (1-0.54) of breast cancer risk, respectively. Secondly, it is worth considering whether it is cost-effective to use high-dose aspirin for the chemical prevention of breast cancer. Comprehensively considering high-dose aspirin intake may cause bleeding complications [23], the optimal aspirin dose for preventing breast cancer may be in the scope of <325 mg per day, 2-7 times/week, along with long-term medication (>5 years).

Though heterogeneity among studies was observed when comparing the highest and lowest frequency as well as the overall use of aspirin, sensitivity analyses indicated that the individual dataset had no significant influence on the overall results. When two studies with NOS ratings of less than 7 were excluded, the pooled RR was similar to that of the overall RR of all included studies. Thus, this meta-analysis's results were robust and confident. Two studies with NOS ratings less than 7 had lower quality in “comparability” and “follow up” than those of high quality studies, which reminded us that these two items were easily neglected in designing or reporting cohort studies. We also performed sensitivity analysis by combining studies at different assessment stages of aspirin exposure, and found the results similar. These results illustrate that the assessment method of aspirin exposure did not have any effect on the conclusions.

Our study has several strengths. It was conducted on 857,831 study participants with an average of 7.6 cases/1,000 person-years of breast cancer from 13 cohort studies. Using this massive database and extended follow-up duration, a stronger statistical power to detect and verify this hypothetical relationship can be generated with our analysis. Additionally, this dose-response design provides a better quantification of the relationship between specified amounts of aspirin intake and the risk of breast cancer, rather than only running a meta-analysis based on the comparison of the extreme categories of medication dosage (high versus low). To the best of our knowledge, this is the first study to explore a threshold effect between the frequency and duration of aspirin intake and breast cancer risk in order to guide the rational application of aspirin in breast cancer prevention.

Meanwhile, several limitations should also be acknowledged. Studies on different age, situ, locality, region and metastasis of breast cancer, estrogen receptor (ER) and progesterone receptor (PR) status are too limited to perform subgroup analysis. Also, although most of the results included in our study come from the completely adjusted models, other biases for the results may have been caused by adjusted models in studies adjusting for different covariates.

In conclusion, an innovative finding in this research is the threshold effect's existence between the frequency and duration of aspirin intake and breast cancer risk in the linear dose-response model. This suggests that the recommended frequency and duration of aspirin intake for breast cancer prevention is 2-7 times/week and >5 years, respectively. The potential benefits and harm associated with aspirin intake in different subgroups should be considered before translating this evidence into clinical applications.

## MATERIALS AND METHODS

### Literature search

A search of Medline, Embase and CENTRAL (Cochrane Library) databases from their inception until October 2016 was conducted to identify potentially eligible studies. We used the string (‘‘aspirin’ OR “acetylsalicylic acid” OR ‘‘ASA’’) AND “breast” AND (“neoplasm” OR “carcinoma” OR “cancer” OR “tumor” OR “adenocarcinoma” OR “sarcoma”). No language restrictions were imposed. The reference lists of all relevant studies were checked for further reports. For our paper, ethical approval was not necessary, as this study is a meta-analysis based on published data. The search strategy is attached in “*Appendix 1*”.

### Inclusion and exclusion criteria

Studies were included if: 1) they were a cohort study; 2) they consisted of an adult population (≥18 years) exposed to different dosages, durations or frequencies of aspirin use with ≥3 quantitatively measured exposure categories; 3) they defined breast cancer incidence as one of their endpoints; or 4) they reported original data including odds ratios (OR), relative risks (RR) or hazard ratios (HR), as well as 95% confidence intervals (CIs). The exclusion criteria for studies was those that were: 1) not full reports; or 2) cross-sectional, case-control designed, reviews, or comments; or 3) animal experimentation was used. The listed references were also checked for additional studies.

### Selection of reports to be studied

To begin, one researcher (LLM) removed the duplicates from the reports, and then scanned the titles and abstracts of the citations (first scanning). This was done with the reference management software EndNote. Two researchers (LLM and ZJC) then cooperatively viewed the full text of all potentially eligible studies. If the literature did not meet the inclusion criteria, or if it met the exclusion criteria, they moved it into the appropriate folder with labels in EndNote X6. Disagreements between the two researchers were discussed and finally resolved by a collaborative effort involving the whole team.

### Exposure and outcome measurements

Aspirin use measure methods (patient-reported or otherwise), assessment periods (at the cohorts' baselines, during follow-up periods or at both periods) and breast cancer screening methods (patient-reported, verified medical records, linkage with the registry or other methods) were summarized from the included cohort studies.

### Data extraction

Two researchers (LLM and ZJC) used the EpiData 3.1 software to extract and enter the information from the final included reports by using a unified structure form. Extracted information included the first author's name, cohort designation, publication year, total number of participants, breast cancer incidence, aspirin use measure, breast cancer screening method, maximum follow-up years, adjustments, confounding factors, aspirin exposure categories (including dosage, frequency and duration), RR, HR or OR with 95% CIs of breast cancer for each aspirin use category.

To clarify the eligibility criteria and to ensure that the criteria could be applied consistently by more than one person, we pilot-tested a draft data abstraction form by randomly including 10 studies before beginning the formal data abstraction. When there was disagreement (i.e. kappa statistic≤0.6), two reviewers discussed and reached agreement. After doing this, we modified and supplemented the original eligibility criteria.

### Methodological quality

The methodological quality of reports was assessed by Newcastle-Ottawa Scale (NOS) [24]. The NOS criteria includes subject selection (scores, 0–4), comparability of subject (scores, 0–2), and exposure or outcome (scores, 0–3) [25], with the total score ranging from 0 to 9. In our meta-analysis, a total score equal to or more than 7 indicated good quality.

### Statistical analysis

The RRs were commonly used across studies. For transformation of RRs, HRs and odds ratios, ORs and HRs were considered RRs. The ORs could be converted into RRs using the formula RR=OR/[(1-P_0_)+(P_0_*OR)], in which P_0_ was the event incidence in the control group [26, 27]. This formula had a limitation of underestimating the variance of the RRs derived from the ORs [28, 29]. Thus, a sensitivity analysis that excluded the one study with this transformation is presented.

First, meta-analysis with RR calculation (the lowest compared with the highest categories of aspirin intake) were used to evaluate the relationship between breast cancer risk and aspirin intake. When there was no heterogeneity, a fixed-effects model was performed to obtain a pooled estimate of effect; otherwise, a random-effects model was applied. Following Greenland and Longnecker [30] and Orsini et al [31], we performed a dose-response analysis of aspirin effects on breast cancer risk. The distribution of cases, person-years and the adjusted RRs with 95% CIs for at least 3 exposure categories were required. We chose the midpoint of the interval when aspirin categories’ intervals were presented. When the upper level for the highest category was open-ended, the exposure doses were calculated as 1.2 times their exposure levels [25, 32]. A potential non-linear dose-response association by modeling dose, frequency or duration of aspirin intake was checked by restricted cubic splines with 3 knots at percentiles 25%, 50%, and 75% of the distribution, respectively. A linear regression model was also built. To test for nonlinearity, a likelihood ratio test was applied to contrast the model with both the linear and the spline terms and the model with the linear term only [33]. If the nonlinear model was invalidated, a linear model was be established to quantify the association of breast cancer risk and aspirin intake.

Statistical heterogeneity was calculated with Cochran's Q (*P*<0.1) and *I^2^* tests (The *I^2^* test represents the percentage of total variation across studies) [34]. To evaluate the robustness of the overall results, sensitivity analyses were used by excluding one study at a time, and estimating the results based on those studies with a score ≥7. This was done to investigate whether study quality had an influence on the overall association. Publication bias was evaluated by funnel plots, and quantified with Begg's and Egger's test [35, 36]. When publication bias existed, we used the trim-and-fill method to evaluate the stability [37]. Stata version 11.0 software (StataCorp LP, College Station, TX, U.S.) was applied to perform all statistical analysis.

## SUPPLEMENTARY MATERIALS AND METHODS


