# Supplementary material for: Aspirin as a potential modality for the chemoprevention of breast cancer: A dose-response meta-analysis of cohort studies from 857,831 participants
Source: Oncotarget. 2017 Mar 17;8(25):40389–401. doi: 10.18632/oncotarget.16315 (PMC5522308; doi:10.18632/oncotarget.16315)
Supplement: Supplementary file 1 [file oncotarget-08-40389-s001.pdf]

Aspirin as a potential modality for the chemoprevention of breast cancer: A dose-response meta-analysis of cohort studies from 857,831 participants

SUPPLEMENTARY MATERIALS AND METHODS

MEDLINE (OVID) search strategy *(October 4, 2016)*

1. exp Aspirin/
2. (aspirin OR acetylsalicylic acid OR ASA).mp
3. 1 or 2
4. exp breast neoplasms/
5. exp breast/
6. exp neoplasms/
7. 5 AND 6
8. (breast$ adj5 (neoplasm$ or cancer$ or tumo?r$ or carcinoma$ or adenocarcinoma$ or sarcoma$ or dcis or ductal or infiltrat$ or intraductal$ or lobular or medullary)).mp.
9. exp mammary neoplasms/
10. (mammar$ adj5 (neoplasm$ or cancer$ or tumo?r$ or carcinoma$ or adenocarcinoma$ or sarcoma$ or dcis or ductal or infiltrat$ or intraductal$ or lobular or medullary)).mp
11. or/4, 7,8-10
12. 3 and 11

EMBASE search strategy *(October 4, 2016)*

1. exp Aspirin/
2. (aspirin OR acetylsalicylic acid OR ASA).mp
3. 1 or 2
4. exp breast neoplasms/
5. exp breast/
6. exp neoplasms/
7. 5 AND 6
8. (breast$ adj5 (neoplasm$ or cancer$ or tumo?r$ or carcinoma$ or adenocarcinoma$ or sarcoma$ or dcis or ductal or infiltrat$ or intraductal$ or lobular or medullary)).mp.
9. exp mammary neoplasms/
10. (mammar$ adj5 (neoplasm$ or cancer$ or tumo?r$ or carcinoma$ or adenocarcinoma$ or sarcoma$ or dcis or ductal or infiltrat$ or intraductal$ or lobular or medullary)).mp
11. or/4, 7,8-10
12. 3 and 11
13. cohort.ti,ot,hw,ab,tn,mf,dm,dv,kw.
14. 12 and 13

CENTRAL (Cochrane Library) search strategy *(October 4, 2016)*

#1 MeSH descriptor: [Aspirin] explode all trees

#2 aspirin or acetylsalicylic acid or ASA:ti,ab,kw (Word variations have been searched)

#3 #1 or #2

#4 MeSH descriptor: [Breast Neoplasms] explode all trees

#5 MeSH descriptor: [Breast] explode all trees

#6 MeSH descriptor: [Neoplasms] explode all trees

#7 #5 and #6

#8 breast$:ti,ab,kw (Word variations have been searched)

#9 neoplasm$ or cancer$ or tumo?r$ or carcinoma$ or adenocarcinoma$ or sarcoma$ or dcis or ductal or infiltrat$ or intraductal$ or lobular or medullary:ti,ab,kw (Word variations have been searched)

#10 #8 and #9

#11 #4 or #7 or #10

#12 #3 and #11.
